# Effect of Topiramate on Weight Status in Young Children With Severe Obesity

**DOI:** 10.1210/jendso/bvaf179

**Published:** 2025-11-11

**Authors:** Mostafa Salama, Doha Hassan, Siobhan Pittock, Aida Lteif, Seema Kumar

**Affiliations:** Division of Pediatric Endocrinology and Metabolism, Department of Pediatric and Adolescent Medicine, Mayo Clinic, Rochester, MN 55905, USA; Department of Pediatric and Adolescent Medicine, Mayo Clinic, Rochester, MN 55905, USA; Department of Pediatric and Adolescent Medicine, Mayo Clinic, Rochester, MN 55905, USA; Division of Pediatric Endocrinology and Metabolism, Department of Pediatric and Adolescent Medicine, Mayo Clinic, Rochester, MN 55905, USA; Department of Pediatric and Adolescent Medicine, Mayo Clinic, Rochester, MN 55905, USA; Division of Pediatric Endocrinology and Metabolism, Department of Pediatric and Adolescent Medicine, Mayo Clinic, Rochester, MN 55905, USA; Department of Pediatric and Adolescent Medicine, Mayo Clinic, Rochester, MN 55905, USA; Division of Pediatric Endocrinology and Metabolism, Department of Pediatric and Adolescent Medicine, Mayo Clinic, Rochester, MN 55905, USA; Department of Pediatric and Adolescent Medicine, Mayo Clinic, Rochester, MN 55905, USA

**Keywords:** topiramate, children, obesity, overweight

## Abstract

**Context:**

Pharmacological options for the treatment of early severe childhood obesity are limited.

**Objective:**

This retrospective case series evaluates the effect of topiramate on weight and cardiometabolic parameters in young children with severe obesity, including those with hypothalamic etiologies.

**Methods:**

This is a retrospective case series including 14 children with severe obesity, who were treated with topiramate prior to age 12 years. Anthropometric and metabolic data were analyzed.

**Results:**

The median age at initiation of topiramate treatment was 10.1 years (range, 7.9-11.2 years) and 64% were boys. At baseline, the median body mass index (BMI) was 149% of the 95th percentile (range, 136%-200%). The median treatment duration was 14 months (interquartile range, 9.5-24 months), with a maximum tolerated dose of 75 mg (range, 50-150 mg). There was a median decrease in BMI% of the 95th percentile of 8% (95% CI, −15 to −1.02; *P* = .042), and a reduction in BMI *z* score by 0.37 (95% CI, −0.66 to −0.05; *P* = .028). The most notable response was in a child with hypothalamic obesity who experienced a 35-point reduction in BMI% of the 95th percentile over 24 months. While 11 of 14 children showed reduction in BMI, 3 had no change or increase in their BMI. No significant changes were observed in cardiometabolic markers or linear growth.

**Conclusion:**

Topiramate monotherapy resulted in significant BMI reductions in young children with severe obesity, including those with hypothalamic obesity. Further prospective randomized controlled trials are warranted to assess its long-term efficacy for obesity management in young children.

Obesity represents a major public health issue, affecting a substantial proportion of children and adolescents, with its prevalence continuing to rise in recent decades [1]. Current estimates indicate that 19.7% of children and adolescents have obesity. This upward trend extends to very young children, with prevalence rates of obesity reaching 12.7% among those aged 2 to 5 years and 20.7% among children aged 6 to 11 years [2]. Furthermore, the proportion of children and adolescents with severe obesity has nearly doubled over the past 25 years, rising from 3.6% in 1999 to 2000 to 7% in 2021 to 2023 [3]. Intensive health behavior and lifestyle treatment, which involves at least 26 hours of structured nutrition, physical activity, and behavior modification over 3 to 12 months, is the most effective behavioral intervention for pediatric obesity. However, its implementation is often challenging, and its benefits for children with severe obesity are generally modest, limited by accessibility [4-6].

The most recent guidelines by the American Academy of Pediatrics recommend that antiobesity pharmacotherapy may be considered as an adjunct to behavioral and lifestyle interventions in children aged 8 years and older with obesity [4]. The US Food and Drug Administration (FDA) has approved several medications for pediatric weight management for adolescents aged 12 and older. However, currently, no medications are approved for the treatment of obesity in children younger than 12, with the exception of setmelanotide, a melanocortin-4 receptor agonist approved for select cases of monogenic obesity and Bardet-Biedl syndrome in children aged 2 and older [7, 8].

Topiramate was approved by the FDA for the treatment of epilepsy in children aged 2 years and older in 1999, and for migraine prophylaxis in those aged 12 years and older in 2014. Topiramate has also been shown to decrease food cravings, binge eating, and appetite [9-11]. These effects are thought to result from its ability to increase γ-aminobutyric acid and decrease glutamate and dopamine secretion in the brain, and also through carbonic anhydrase inhibition [9, 10]. Topiramate monotherapy was associated with a 4.9% decrease in body mass index (BMI) change over a 6-month period in adolescents with severe obesity [12]. Several other smaller studies have also demonstrated modest reductions in BMI in children with topiramate monotherapy [13-15]. However, existing data on the weight loss effects of topiramate are primarily derived from studies conducted in adolescents or in the context of epilepsy management. Data on the efficacy and safety of topiramate monotherapy particularly in children younger than 12 years remain limited [13].

In this study, we conducted a retrospective evaluation of the effect of topiramate on weight and cardiometabolic parameters in children aged 12 years or younger with severe obesity.

## Materials and Methods

The study included 14 pediatric patients with severe obesity, defined as BMI at or above 120% of the 95th percentile for age and sex, who were treated with topiramate for weight management. All participants were evaluated at the Pediatric Endocrinology Clinic of Mayo Clinic in Rochester, Minnesota. Research authorization status was obtained prior to study enrollment, and the study was approved by the Mayo Clinic Institutional Review Board.

Topiramate was used for weight management due to the absence of FDA-approved pharmacotherapies for obesity in children younger than 12 years. Moreover, all participants had severe obesity prior to treatment, and lifestyle interventions alone had not yielded sustained weight reduction. A subset of the children had hypothalamic obesity, further complicating obesity management.

Data were obtained retrospectively from electronic medical records, including demographic and anthropometric variables (age, sex, race, ethnicity, body weight, height, BMI, BMI% of the 95th percentile, BMI *z* score, and height percentiles). Measurements were taken at baseline (prior to the initiation of topiramate) and during treatment until medication discontinuation or data collection. BMI% of the 95th percentile was used to assess changes in BMI status as the traditional metrics of BMI percentile and BMI *z* score lose sensitivity and accuracy at the upper extremes of BMI [16, 17]. Unlike percentiles or *z* scores, BMI expressed as a percentage of the 95th percentile provides a linear, interpretable, and responsive measure of obesity severity and change over time, making it the recommended standard in current American Academy of Pediatrics guidelines [4].

Additional clinical information collected included the reported age at onset of weight gain, BMI% of the 95th percentile 1 year before treatment, obesity-related comorbidities, genetic testing results, epilepsy diagnosis, and brain magnetic resonance imaging findings. Cardiometabolic parameters including fasting glucose, glycated hemoglobin A_1c_ (HbA_1c_), lipid profile, blood pressure, and alanine transaminase (ALT) were also measured both before and during treatment. Data were managed using REDCap (Research Electronic Data Capture).

### Statistical Methods

Histograms and Q-Q plots were used to assess the distribution of continuous variables. Descriptive statistics were employed to summarize the baseline characteristics of the study participants. Categorical variables were reported as frequencies and percentages, while continuous variables were summarized using medians and interquartile ranges (IQRs) due to nonnormal distributions.

The Wilcoxon signed rank test was employed to conduct paired analysis of anthropometric and cardiometabolic parameters before and after treatment with topiramate. A 2-sided hypothesis was tested, with the null hypothesis stating that the median difference between pretreatment and posttreatment values was zero. This methodology was used to evaluate changes in BMI% of the 95th percentile, BMI *z* score and, height percentile as well as cardiometabolic markers, including fasting glucose, HbA_1c_, lipid parameters, and ALT. Simple linear regression models were performed to examine whether baseline age at medication start or medication dose were associated with changes in BMI% of the 95th percentile, with change in BMI% of the 95th percentile specified as the dependent variable.

All statistical analyses were performed using BlueSky Statistics version 10.x. A *P* value less than .05 was considered statistically significant.

## Results

The study included 14 children with severe obesity, with a median age of 10.1 years (range, 7.9-11.2 years) (Table 1). Median baseline BMI% of the 95th percentile was 149% (range, 136%-200%), and the median BMI *z* score was 3.19 (range, 2.63-5.14). Nine children were boys (64%), 12 (86%) were not Hispanic or Latino, and 14 (100%) were White. Prior to treatment, 12 out of 14 participants had class III obesity (BMI ≥140% of 95th percentile). Two children had a diagnosis of epilepsy. Three children were diagnosed with hypothalamic obesity; 2 had a history of craniopharyngioma treated with surgical resection, followed by hyperphagia and multiple pituitary hormone deficiencies, and 1 child had septo-optic dysplasia–increased appetite with panhypopituitarism on hormone replacement. The hormone deficiencies in all 3 children were being appropriately treated. The median age of obesity onset for the entire cohort was 4.7 years (range, 1.0-9.1 years). Eight children had dyslipidemia, 5 had obstructive sleep apnea, and 4 had metabolic dysfunction–associated steatotic liver disease.

**Table 1.** Baseline characteristics of study participants

Variable	No. of observations	Percentage
Sex	14	
Male	9	64%
Female	5	36%
Ethnicity	14	
Not Hispanic or Latino	12	86%
Hispanic or Latino	2	14%
Race		
White	14	100%
Insurance	14	
Private	11	79%
Public	3	21%
Obesity-related comorbidities	12	
Dyslipidemia	8	57%
MASLD	4	29%
Hypothalamic obesity	3	22%
Sleep apnea	5	36%
Elevated blood pressure	2	14%
Adverse events	2	14%
Somnolence	1	
Fatigue	1	

Variable	No. of observations	Median (IQR)	Range
Age of medication start, y	14	10.12 (8.60-10.68)	7.90-11.20
Age of obesity onset, y	14	4.70 (3.85-5.38)	1.00-9.10
Treatment duration, mo	14	14.00 (9.25-24.00)	3.00-24.00
Topiramate maximum tolerated dose, mg/d	14	75.00(50.00-100.00)	50.00-150.00
BMI% of 95th percentile	14	149.00 (141.25-170.00)	136.00-200.00
BMI *z* score	14	3.19 (2.89-4.05)	2.63-5.14
HbA_1c_, %	13	5.20 (5.10-5.40)	4.80-5.60
Fasting blood glucose, mg/dL	12	90.00 (86.00-93.50)	80.00-96.00
TC, mg/dL	12	171.50 (154.75-190.25)	133.00-218.00
LDL-C, mg/dL	12	82.50 (75.25-100.50)	64.00-130.00
HDL-C, mg/dL	12	43.00 (33.25-48.75)	27.00-76.00
Non–HDL-C, mg/dL	12	116.50 (111.25-143.50)	82.00-170.00
TGs, mg/dL	12	172.00 (133.75-251.00)	80.00-437.00
ALT, U/L	12	40.00 (26.50-68.75)	16.00-216.00

Abbreviations: ALT, alanine transaminase; BMI, body mass index; HbA_1c_, glycated hemoglobin A_1c_; HDL, high-density lipoprotein cholesterol; IQR, interquartile range; LDL-C, low-density lipoprotein cholesterol; MASLD, metabolic dysfunction–associated steatotic liver disease; TC, total cholesterol; TGs, triglycerides.

Brain magnetic resonance imaging was performed in 10 out of 14 participants. As noted, 3 children had known abnormalities causing hypothalamic obesity (craniopharyngioma status post resection and septo-optic dysplasia). One child had a small Rathke cleft cyst in the pituitary gland that was not associated with clinical symptoms. No other significant imaging abnormalities were seen.

Five patients had previously undergone genetic testing for monogenic obesity. Among them, 1 had 5 heterozygous variants of uncertain significance (VUS) in the genes *IFT74*, *PCNT*, *SEMA3C*, *SEMA3F*, and *VPS13B*, associated with autosomal recessive conditions. One patient had a heterozygous VUS in *SEMA3G*, a gene linked both to autosomal dominant and recessive inheritance patterns. Additionally, heterozygosity for recessive VUS in the *ADCY3* and *TTC8* genes were noted in another patient. One patient with generalized hypotonia had whole-genome sequence analysis that revealed a mitochondrial VUS in the *MT-RNR1* gene at location (m.859T > C), and one had negative results.

**Figure 1.**
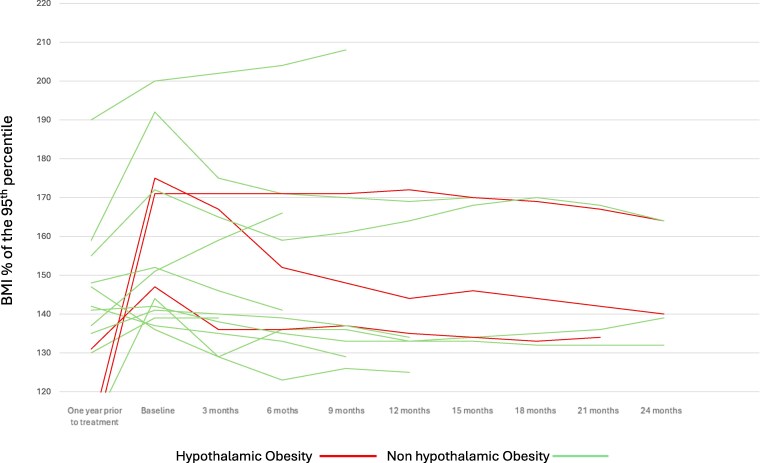
Longitudinal changes in body mass index percentage (BMI%) of the 95th percentile before and during treatment with topiramate in 14 pediatric patients with severe obesity. Each line represents an individual patient's BMI% of the 95th percentile over time, measured at regular intervals following initiation of topiramate.

Median BMI% of the 95th percentile had increased by 12 points (95% CI, 5-34; *P* = .005) in the year prior to initiation of topiramate (Fig. 1). The median maximum tolerated dose of topiramate was 75 mg (range, 50-150 mg), with a median treatment duration of 14 months (IQR, 9.5-24 months). Five children continued treatment for 24 months, and 9 of the 14 remained on treatment for at least 12 months.

After starting topiramate, the median BMI% of the 95th percentile dropped by 8 points (95% CI, −15% to −1.02%; *P* = .042), and the BMI *z* score decreased by 0.37 (95% CI, −0.66 to −0.05; *P* = .028). One child had no change in his BMI, and 2 children continued to have an increase in BMI. One child with hypothalamic obesity had the greatest response, with a 35-point drop in his BMI% of the 95th percentile over 24 months. Of the 2 remaining children with hypothalamic obesity, 1 experienced a reduction in BMI% of the 95th percentile by 13 points following 21 months of treatment. The second child's BMI showed a modest 7-point reduction in BMI% of the 95th percentile after 24 months of therapy; notably this child had previously exhibited an increase of 66 points in BMI% of the 95th percentile during the year preceding the initiation of treatment. There was significant variability in response to topiramate in our cohort. While 11 children had a decrease in their BMI% of the 95th percentile (range, −5% to −35%) relative to baseline prior to treatment, 3 children exhibited no response. One child had no change in his BMI% of the 95th percentile, while the other 2 children had an increase in their BMI% of the 95th percentile by 8 and 15 points, respectively (Fig. 1). Additionally, 3 patients exhibited an initial decrease in BMI% of the 95th percentile followed by a rebound increase in BMI% of the 95th percentile while on treatment (see Fig. 1).

In univariable linear regression, age at medication initiation was not a statistically significant predictor of change in BMI% of the 95th percentile following topiramate treatment (*P* = .18). For age-based analyses, participants were also stratified into 2 groups using 10 years, the cohort's median age, as the cutoff, yielding 7 patients per group. Among children younger than 10 years, the median reduction in BMI% of the 95th percentile was 11 points (95% CI, −28% to −6.6%; *P* = .036). Children aged 10 years and older demonstrated a median reduction of 7 points (95% CI, −10 to 11.5; *P* = .6). Comparison between these groups showed no statistically significant differences in the reduction in BMI (median difference 6 points [95% CI, −26 to 3]; *P* = .16). Additionally, there was no difference in BMI% of the 95th percentile response by sex (*P* = 0.841), medication dose (*P* = .537), or hypothalamic obesity etiology (*P* = .184).

No statistically significant changes in any cardiometabolic outcomes parameters were observed following topiramate treatment (Table 2). Adverse events occurred in 2 children: One developed somnolence at a dose of 50 mg after 1 month, which resolved spontaneously after the dose was shifted to bedtime; the other reported fatigue at 75 mg after 4 months leading to a reduction of the dose to 50 mg.

**Table 2.** Changes in anthropometric and cardiometabolic parameters after topiramate

Variable	No. of participants with data	Median (95% CI)	Range	*P*
BMI % of 95th percentile	14	−8 (−15 to −1.02)	−35 to 15	.042
BMI *z* score	14	−0.37 (−0.66 to −0.05)	−1.56 to 0.55	.028
Height percentile	11	−0.03 (−4.0 to 3.17)	−7 to 9	.683
TC, mg/dL	10	−10 (−28.8 to 10.5)	−46 to 26	.232
LDL-C, mg/dL	9	−3 (−22 to 14)	−36 to 31	.779
HDL-C, mg/dL	9	0 (−8.5 to 5)	−10 to 6	.670
Non–HDL-C, mg/dL	9	−9 (−23.5 to 12.5)	−38 to 14	.635
TGs, mg/dL	9	−4.5 (−56.5 to 76)	−94 to 159	.820
ALT, U/L	10	−3 (−34.5 to 2)	−65 to 20	.102
Fasting blood glucose, mg/dL	11	−2 (−5 to 3.5)	−9 to 14	.422
HbA_1c_, %	10	0.05 (−0.1 to 0.15)	−0.3 to 0.2	.523

Abbreviations: ALT, alanine transaminase; BMI, body mass index; HbA_1c_, glycated hemoglobin A_1c_; HDL-C, high-density lipoprotein cholesterol; LDL-C, low-density lipoprotein cholesterol; TC, total cholesterol; TGs, triglycerides.

Growth velocity in children with hypothalamic obesity may be influenced by factors such as puberty, growth hormone status, and craniospinal radiation. Therefore, the effect of topiramate on linear growth was assessed in the 11 children without hypothalamic obesity. No statistically significant difference was observed in height percentiles following topiramate treatment (median difference: −0.03%; 95% CI, −4.0% to 3.17%; *P* = .683).

## Discussion

This study examines the outcomes of topiramate monotherapy for weight management in 14 children younger than 12 with severe obesity. Treatment with topiramate was associated with a reduction in median BMI% of the 95th percentile by 8 points, and in median BMI *z* score by 0.37. Improvement in BMI was noted in 3 patients with hypothalamic obesity. To our knowledge, this is the largest study to report the outcomes of topiramate monotherapy for children younger than 12 with severe obesity. Heterogeneity in response was noted as improvement in BMI with topiramate monotherapy was noted in 11 of the 14 children in our study, whereas the other 3 children had no change or increase in their BMI.

### Efficacy of Topiramate in Children With Severe Obesity

Our results of improvement in BMI with topiramate monotherapy are similar to previous, smaller studies. Berman and colleagues [13] reported a reduction in BMI% of the 95th percentile by 12% (range, −5% to −18%) in a case series of 5 children with a mean age of 10 years and 3 months. The case series had a 16-week follow-up, whereas our study included follow-up extending up to 2 years. In contrast to our findings and those of Berman et al [13], no weight loss was found in another retrospective study by Czepiel et al [14] in 17 children aged 5 to 12 years (reported change in BMI *z* score of 0.03 [95% CI: −0.1 to 0.1]; *P* > .1). However, topiramate was associated with modest weight loss in adolescents [14]. In one study involving 50 children with epilepsy (mean age, 8.09 ± 5.46 years) with normal BMI at baseline who were treated with topiramate for seizure management, the topiramate group demonstrated a mean BMI change of −0.81 (*P* = .019), while the control group exhibited an increase of +0.46 (*P* = .023). Notably, weight loss was observed in 21 of the 50 children (42%) receiving topiramate [18].

The weight loss effect of topiramate monotherapy in children younger than 12 years is similar to that reported in older adolescents. Mean BMI% reduction of 4.9% was reported by Fox et al [12] in a study of 28 adolescents (mean age, 15.2 ± 2.5 years) treated with topiramate in addition to lifestyle interventions for 6 months. Similarly, topiramate monotherapy was associated with a reduction in BMI% of the 95th percentile by 9.3% after 1 year of treatment in a large retrospective cohort study involving 282 adolescents with a mean age of 12.7 years and severe obesity. However, unlike our study, adolescents with known hypothalamic obesity were excluded [19].

Topiramate, approved for seizures and migraine prophylaxis, has been associated with appetite suppression, reduced food cravings, and weight loss, which led to its use off-label as an antiobesity medication. While the precise mechanisms are unclear, several pathways have been proposed. Animal studies suggest that topiramate may enhance the expression of anorexigenic neuropeptides, including pro-opiomelanocortin, thyrotropin-releasing hormone, and corticotropin-releasing hormone, within the hypothalamus, thereby modulating the melanocortin-4 receptor pathway and reducing food intake [10]. Other possible mechanisms include inhibition of voltage-gated sodium channels, modulation of glutamatergic transmission, inhibition of carbonic anhydrase, and potentiation of γ-aminobutyric acid activity, all linked to appetite control and energy balance [9, 10].

### Effect of Topiramate in Hypothalamic Obesity:

In our study, 3 children with hypothalamic obesity demonstrated improvement in BMI following treatment with topiramate, with either significant reductions in BMI or stabilization of their BMI trajectory, suggesting that topiramate may have therapeutic potential in this challenging subgroup. There is limited evidence supporting the use of monotherapy with topiramate or combination therapy with phentermine and topiramate in individuals with syndromic obesity (Prader-Willi syndrome and Bardet-Biedl syndrome) [20-22]. Currently, a clinical trial is under way to assess the efficacy of phentermine/topiramate extended-release therapy in children aged 12 years and older with hypothalamic obesity (NCT06299891).

### Effect of Topiramate on Cardiometabolic Parameters

Evidence suggests that topiramate may have a favorable effect on cardiometabolic health, including improvements in lipid profiles and metabolic dysfunction–associated steatotic liver disease [23, 24]. However, in our cohort, we did not observe statistically significant changes in cardiometabolic biomarkers following treatment. Importantly, no worsening of metabolic status was noted during the study period. The absence of detectable changes may be attributable to the limited sample size, which reduced the power to detect significant pretreatment and posttreatment differences.

### Topiramate Side Effects, Weight Loss Durability, and Study Limitations

The side effects of topiramate are generally dose dependent and can include cognitive dysfunction, paresthesia, kidney stones, metabolic acidosis, glaucoma, and teratogenicity [13, 25]. In our cohort, the only reported side effects in 2 children were somnolence and fatigue. Some studies report a slight reduction in linear growth velocity with topiramate, whereas others show no effect [18, 26]. In our study, we did not identify any negative effect of topiramate on children's linear growth.

This study has several notable strengths. It evaluates the effects of topiramate for weight management in children younger than 12, including those with hypothalamic obesity, a group not previously studied. The extended follow-up duration, with some patients followed for up to 2 years, offers valuable insights into the durability of treatment effects and BMI trajectory over time. Additionally, the inclusion of a younger age group helps address a critical gap in the literature regarding early interventions for severe obesity with limited pharmacological options.

One limitation of our study is the short duration of follow-up. The youngest child in our cohort, who was aged 7 years and 10 months, experienced a reduction in BMI% of the 95th percentile by 5 points after 24 months of treatment with topiramate; however, a slight rebound increase in BMI was observed after 15 months. A similar pattern was noted in 4 other children, in whom an initial decline in BMI occurred during the early months of treatment followed by stabilization or an increase thereafter. This trend raises questions about the long-term sustainability of topiramate's weight-lowering effects. We also do not have data on BMI trends after the discontinuation of topiramate in those that had a response as these patients were still receiving the medication at the time of the data collection. Significant heterogeneity was noted with regard to weight loss among the patients, and the small sample size precluded our ability to identify patient characteristics that predict weight loss with topiramate.

The retrospective study design and small sample size limit the statistical power to detect subtle changes, particularly in cardiometabolic outcomes. Additionally, data on adherence, diet, physical activity, lifestyle interventions, and pubertal status were not available, all of which could potentially influence BMI and cardiometabolic outcomes. The absence of a control group limits our ability to make causal inferences regarding the effects of topiramate. Also, as this study was conducted in a tertiary care center, referral and prescribing practices may introduce bias and the results may not reflect general pediatric practice. The cohort was predominantly White and non-Hispanic, and the single institution experience may restrict the generalizability of the findings to broader, more heterogeneous pediatric populations.

## Conclusion

This study provides initial findings on the potential role of topiramate in managing severe obesity in young children younger than 12 years, including individuals with hypothalamic obesity. Several children achieved weight stabilization or improvement, particularly among those with hypothalamic obesity, who generally have fewer treatment options. The treatment was well tolerated, with no notable adverse effects on growth observed during the study period. These findings suggest that topiramate may be a viable pharmacologic adjunct for severe pediatric obesity in selected cases, particularly where lifestyle interventions have failed. Furthermore, the data highlight the importance of conducting larger, prospective randomized clinical trials to further assess the long-term efficacy, safety, and sustainability of topiramate therapy for pediatric obesity.

## Data Availability

Original data generated and analyzed during this study are included in this published article or in the data repositories listed in “References.”

## Abbreviations

ALT: alanine transaminase
BMI: body mass index
FDA: US Food and Drug Administration
HbA_1c_: glycated hemoglobin A_1c_
VUS: variant of uncertain significance
